# Evaluation of Bone Mineral Density, Serum Osteocalcin, and Osteopontin Levels in Postmenopausal Women with Type 2 Diabetes Mellitus, with/without Osteoporosis

**DOI:** 10.1155/2022/1437061

**Published:** 2022-02-14

**Authors:** Ali B. Roomi, Abdul-Hassan Mahdi Salih, Sarmad D. Noori, Wassan Nori, Saba Tariq

**Affiliations:** ^1^Unviersity of Thi-Qar, Nasiriyah, Thi-Qar 64001, Iraq; ^2^Biochemistry and Biological Engineering Research Group, Scientific Research Center, Al-Ayen University, Nasiriyah, Thi-Qar 64001, Iraq; ^3^Department of Physiology, College of Medicine, Unviersity of Thi-Qar, Nasiriyah, Iraq; ^4^Department of Pharmaceutical Chemistry, College of Pharmacy, Al-Ayen University, Nasiriyah, Thi-Qar, Iraq; ^5^Department of Obstetrics and Gynecology, College of Medicine, Mustansiriyah University, Baghdad, Iraq; ^6^Department of Pharmacology, University Medical & Dental College, The University of Faisalabad, Faisalabad 38000, Pakistan

## Abstract

**Objective:**

Osteoporosis (OP) is a worldwide ailment; we aim to establish new biomarkers in diagnosis by determining the levels of serum osteocalcin and osteopontin along with bone mineral density (BMD) and lumbar T-score, in postmenopausal women with type 2 diabetes mellitus (T2DM) with or without OP.

**Methods:**

This observational study included 160 postmenopausal women who were an attendee at outpatient clinics in Al-Hussein Hospital, Thi-Qar province; subdivided into 3 groups based on their T-score testing: Group I (*n* = 40) comprised postmenopausal women without T2DM as controls, Group II (*n* = 60) comprised postmenopausal women with T2DM but without OP, and Group III (*n* = 60) comprised postmenopausal women with T2DM with OP. The dual-energy X-ray absorptiometry was used to measure the BMD (total body, lumbar spine, and femoral) and T-score for lumbar spine and femoral. Glycosylated hemoglobin (HbA1c), fasting blood glucose (FBG), osteocalcin, and osteopontin levels were assessed in all three groups.

**Results:**

Compared with controls, Group III demonstrated significantly lower BMD (total body, lumbar spine, and femoral), T-score for lumbar spine and femoral, serum osteocalcin, and osteopontin levels than Group II and Group I (*P* < 0.001). FBG and HbA1c levels were significantly higher in Group III than in Groups I and II (*P* < 0.001). A negative correlation was proved between HbA1c levels with BMD, osteocalcin levels, and osteopontin levels in the three groups.

**Conclusions:**

Iraqi postmenopausal women with T2DM had a significantly lower bone mineral density, serum osteocalcin, and osteopontin levels. These results may serve as adjuvants in screening for OP, particularly among diabetic patients.

## 1. Introduction

Osteoporosis (OP) is a common bone illness that affects people worldwide and is characterized by reduced bone mineral density (BMD) and deteriorated bone tissues, making the bones fragile [1, 2]. Diabetes mellitus (DM) is a metabolic condition characterized by elevated blood glucose levels due to insulin resistance, deficiency, or both [3]. Type 2 DM (T2DM), characterized by hyperglycemia, accounts for about 90% of all diabetes cases worldwide [4]. Patients with type 1 DM have a lower BMD whereas those with T2DM are at a higher risk of experiencing a bone fracture, given their high or average BMD as measured by dual-energy X-ray absorptiometry (DXA) [4, 5]. Other causes that contribute to the development of fractures in patients with T2DM include sarcopenia, impaired balance due to peripheral neuropathy, diabetic retinopathy, and medications used in diabetes [6, 7].

The postmenopausal period is often related to OP because of the hypoestrogenic condition induced by ovarian failure [3, 8]. OP is better prevented than treated; the enhancement of public understanding of its consequences and risks by the media and community newsletter agencies has contributed to a fair reduction in many countries that have embraced this approach [9–11].

Osteocalcin (OC) is a small bone-specific noncollagen protein found chiefly in the bone and is produced by osteoblasts. OC is a sensitive marker of bone formation. OC is carboxylated and mostly combined within the bone extracellular matrix; however, a small proportion is released into the circulation where OC plays a role in fat and glucose metabolism [12]. Osteopontin (OPN) is a noncollagenous matrix protein in bone tissues and is involved in bone remodeling and stimulates bone resorption [11, 13]. The association of OP biomarkers with T2DM is well known, but studies from Thi-Qar, Iraq, are few. This is a leading study in south Iraq, Thi-Qar governorate, that aims to evaluate the BMD and serum OC and OPN levels in postmenopausal women with T2DM along with selected demographic criteria.

## 2. Materials and Methods

In this observational study, we included 210 postmenopausal women who attended Al-Hussein Teaching Hospital and private clinics in Thi-Qar Province, Iraq. After the exclusions, 160 postmenopausal women remained. The volunteers were sampled between November 2019 and March 2020. All of them underwent a physical exam, blood sampling, and bone mineral density measurement. Volunteers in this study were postmenopausal women based on the World Health Organization definition for menopause including those who had their last menstrual period at least one year ago [14]. Females with rheumatic disease, malignancy, gout, hypertension, history of smoking, history of alcohol consumption, and abnormal renal, liver, or thyroid function were excluded. Similarly, females receiving medications that impact bone metabolism (bisphosphonates, strontium translate, selective estrogen receptor modulators, calcitonin, cathepsin-K inhibitors, and parathyroid hormone) were also excluded. DXA scan was used to measure the T-score value at the lumbar spine (LS: L1–L4) and femoral neck (FN). All scans were conducted using the DXA scanner by DEXXUM3 (Osteosys Co. Ltd., Korea) at Al-Hussein Teaching Hospital. T-score value of 0 to 1 was regarded as normal BMD, −1 to −2.5 was considered as osteopenia, and ≤−2.5 was estimated as OP [15]. The participants in this study were divided into 3 groups: Group I, 40 postmenopausal women as controls; Group II, 60 postmenopausal women with T2DM but without OP; and Group III, 60 postmenopausal women with T2DM with OP based on their T-score.

A five mL fasting blood sample was collected from each participant and divided into 2. A two mL portion was placed in an EDTA tube and was used to measure glycated hemoglobin (HbA1c), and the other three mL was allowed to clot and then centrifuged at 3600 rpm for 10 min. The serum was separated from the coagulants and stored at −20°C. Serum OC and OPN levels were estimated using an enzyme-linked immunosorbent assay (ELISA) kit provided by Demeditec Diagnostics GmbH, Germany. Serum fasting blood glucose (FBG) concentration was estimated according to the Copper assay kit (Randox, England) by using the spectrophotometer. The precipitate was evaluated by measuring the red (total hemoglobin) and the blue (glycosylated hemoglobin) color intensity with the NycoCard Reader (Axis-Shield Co., Norway).

## 3. Statistical Analysis

All statistical analyses were performed by using the program IBM (SPSS) version 26. The data in this study were shown as mean ± SD. One-way ANOVA test was used to compare the three study groups. Significance was set for a *P* value of <0.01. Pearson's correlation coefficient tested the correlation between HbA1c as a dependent variable and BMD, OC, and OPN in three groups. The correlation coefficient is positive if the 2 variables under consideration tend to increase or decrease together, and the larger the value, the closer the association. Conversely, the correlation coefficient is negative if an increasing value of 1 variable tends to go with a decreasing value for the other and vice versa.

## 4. Results

The general characteristics of postmenopausal women are shown in Table 1. Markers such as BMD LS (0.57 ± 0.10 g/cm^2^), T-score (−3.60 ± 1.80), serum OC (19.30 ± 1.72 ng/mL), and OPN (14.2 ± 1.40 ng/mL) were significantly lower in Group III compared to BMD (0.78 ± 0.10), T-score (−2.01 ± 0.90), serum OC (24.40 ± 2.32), and OPN (18.10 ± 2.01) in Group II and BMD (0.98 ± 0.12), T-score (0.92 ± 0.12), serum OC 924.40 ± 2.61, and OPN (19.40 ± 2.60) in Group I, with *P* < 0.001.

In Group III, FBG (8 ± 1.4 mmol/L) and HbA1c (8.01 ± 2.01%) were significantly higher compared to those in Group II (5.01 ± 1.20 and 7.12 ± 1.60) and Group I (4.10 ± 1.01 and 5.01 ± 1.41), respectively. A significantly lower BMD LS, BMD FN, and total body BMD were observed in Group III women compared with those in Groups I and II. Serum FBG and HbA1c were highest in Group III. FBG was significantly lower in Group II compared with Group I. HbA1c level, taken as a dependent marker, was calculated by linear regression analysis against T-score, OC, and OPN as independent markers. The results of HbA1c showed a significant negative correlation with BMD, OC, and OPN in three groups as illustrated in Table 2.

## 5. Discussion

T2DM is a chronic metabolic disease that has been associated with various risk factors. Diabetes and OP frequently coexist in elderly individuals; this unhealthy alliance affects the quality of life. The incidence of osteoporosis continues to rise along with fragility fractures [16]. The objective of the study was to see the relationship between BMD, OC, and OPN levels in postmenopausal females with type 2 DM with and without osteoporosis.

We found that BMD and serum levels of OC and OPN were significantly lower in postmenopausal females with type 2 DM with OP. Similar to our results, another study found a decreased level of OC in the postmenopausal osteoporotic group [17]. In contrast to our results, another study found a significant negative association of serum OC with BMD [18]. However, the major difference was that our patients were diabetic and there could be possible interaction of OC with glucose metabolism showing some energy metabolism disorders in our females.

We also could not find any association of BMI with OP. Similar to our results, few other studies could not establish any association of BMI with OP [19, 20]. In contrast, evidence also suggests that increased BMI has a protective role against OP as an increase in mechanical loading of bones leads to increased bone mass [21].

T-score LS and T-score FN in Group III were significantly lower especially in postmenopausal women with T2DM, in line with few other studies which had shown significantly reduced BMD is in patients suffering from T2DM [22]. In this study, the mean HbA1c and serum FBG were significantly increased in patients with OP. Our results support the idea that chronic hyperglycemia reduces bone quality by inhibiting OC secretion and increasing bone resorption. Another point to consider is diabetes-related comorbidities as diabetic retinopathy and peripheral neuropathy are responsible for increased falls and thus increased fractures. There is conflicting evidence that there is a relationship between insulin or other oral medication and raised risk of osteoporosis [6–9]. Moreover, antidiabetic therapy was blamed to increase OP risk since insulin increases fall by hypoglycemic attacks [6, 22]. The use of thiazolidinediones has been associated with increased fracture risks in both men and women with T2DM as it inversely affects the bone quality by suppressing the differentiation of mesenchymal stem cells to osteoblasts in favor of differentiation to adipocytes [6, 23].

OC is a bone-derived hormone which is involved in bone formation and calcium homeostasis. It protects against high-fat-induced obesity and insulin resistance. It had been observed that patients with OP showed a substantial decrease in serum OC, and these levels were positively related to poor regulated DM and are inversely related to FBG. Moreover, lower serum OC was associated with increased mortality in women with T2DM [22–24]. This study confirmed an inverse correlation between HbA1c and OC levels in line with another study who reported a significant inverse relation between serum OC level and forearm BMD in patients with knee osteoarthritis (KOA) [25].

Impaired bone formation and uncoupling of bone turnover was evident even in postmenopausal patients with rheumatoid arthritis and increased the risk of hip fracture [26]. There was a significant decrease in the T-score of the hip and serum levels of OC in postmenopausal Egyptian women with obesity [27]. The last conclusions were consistent with our findings, where an inverse correlation was proven between BMD and OC levels.

OPN, a multifunctional protein primarily associated with bone metabolism, bone remodeling, and bone turnover proved to be inversely related to HbA1c. OPN levels tend to rise as glucose tolerance deteriorates in humans [13]. Earlier studies support our findings assuming a positive correlation with HbA1c, FBG, 2-hour plasma glucose, and OPN [13]. Furthermore, OPN was found to be related to progressive joint damage in KOA and serves as a biochemical marker for determining disease severity [28].

In Iraqi postmenopausal females, the incidence of OP increased to 22.8% [29]. This study highlights the need to establish risk prediction models to avoid consistently underestimating the risk of OP-related fractures in patients with diabetes. There is an emerging need for fracture prevention approaches in patients with T2DM. Several Arab countries have acknowledged the problem and have considered taking initiatives to minimize its dramatic impact on the well-being of women and health costs. The introduction of several effective pharmaceutical agents to treat those at high risk has yielded a promising basis for conducting public health education [9]. The decline in hip fracture in Lebanon could be because of multiple factors: updating the first Lebanese OP assessment and therapeutic guidelines in 2007 along with the pervasive use of calcium and vitamin D supplements. OP rates in Morocco and Kuwait remain stable, slowly rising from 2009 to 2012, and can be ascribed to an increase in the population group aged ≥50 years [10]. The United States recently noted a higher incidence of OP; a decline in DXA screening could be a major factor, along with a decline in bisphosphonate drug prescription. The US Food and Drug Administration updated amended labeling for risk of OP and atypical femur fractures [30–32].

Limitations of this study include the small sample size used as the hospital was transformed into a coronavirus disease 2019 emergency hospital during the pandemic. Moreover, the socioeconomic status of the participants should have been taken into account so that more access to earlier diagnostic and preventive strategies would have presented those with a degree of osteopenia rather than those with OP. The novelty of this work lies in the fact that it tackled the issue of OP despite the lack of public knowledge of its serious complications, the limited funding, the poor socioeconomic status of many Thi-Qar residents, and the high cost of DXA scanning along with the lack of vitamin and mineral supplements.

## 6. Conclusions

This study has proven that Iraqi postmenopausal women in Thi-Qar Province had a reduced bone mineral density along with a significant reduction in serum OPN and OC in postmenopausal women with diabetes. These parameters may be used as adjuvants in screening for the risk of OP with particular emphasis on patients with diabetes to ensure that elderly Iraqi women grow crowned with health.

## Figures and Tables

**Table 1:** Characteristics of postmenopausal women according to groups.

Parameter mean ± SD	Group I (*n* = 40)	Group II (*n* = 60)	Group III (*n* = 60)	*P* value
Age (years)	58.20 ± 6.01	58.70 ± 5.01	57.91 ± 5.40	0.22
Menopause duration (years)	9.21 ± 4.61	8.92 ± 4.22	9.01 ± 4.40	0.14
T2DM duration (years)	—	7.21 ± 5.3	7.81 ± 5.60	0.12
BMI (kg/m^2^)	24.90 ± 1.01	26.10 ± 2.61	27.01 ± 2.40	0.19
Total body BMD (g/cm^2^)	0.91 ± 0.14	0.80 ± 0.10	0.60 ± 0.12	<0.001^*∗*^
BMD LS (g/cm^2^)	0.98 ± 0.12	0.78 ± 0.10	0.57 ± 0.10	<0.001^*∗*^
BMD FN (g/cm^2^)	0.61 ± 0.12	0.52 ± 0.14	0.43 ± 0.17	<0.001^*∗*^
T-score LS	0.92 ± 0.12	−2.01 ± 0.90	−3.60 ± 1.80	<0.001^*∗*^
T-score FN	0.83 ± 0.16	−1.98 ± 0.13	−3.94 ± 1.15	<0.001^*∗*^
Fasting glucose (mmol/L)	4.10 ± 1.01	5.01 ± 1.20	8.01 ± 1.41	<0.001^*∗*^
HbA1c (%)	5.01 ± 1.41	7.12 ± 1.60	8.01 ± 2.01	<0.001^*∗*^
OC (ng/mL)	24.40 ± 2.61	24.40 ± 2.32	19.30 ± 1.72	<0.001^*∗*^
OPN (ng/mL)	19.40 ± 2.60	18.10 ± 2.01	14.2 ± 1.40	<0.001^*∗*^

BMI, body mass index; BMD, bone mineral density; LS, lumbar spine; FN, femoral neck; HbA1c, hemoglobin A1c; OC, osteocalcin; OPN, osteopontin. ^*∗*^indicates significance.

**Table 2:** The correlations of HbA1c level versus BMD, OC, and OPN in the studied subgroups.

Studied groups	BMD		OC		OPN	
	*r*	*P* value	*r*	*P* value	*r*	*P* value
Group I	−0.312	<0.001^*∗*^	−0.381	<0.001^*∗*^	−0.341	<0.001^*∗*^
Group II	−0.345	<0.001^*∗*^	−0.391	<0.001^*∗*^	−0.347	<0.001^*∗*^
Group III	−0.478	<0.001^*∗*^	−0.454	<0.001^*∗*^	−0.431	<0.001^*∗*^

^*∗*^Correlation is at significant level at 0.001.

## Data Availability

The data will be provided upon reliable reason from the corresponding author.
